# Triple barcoding for a hyperparasite, its parasitic host, and the host itself: a study of *Cyclocotyla bellones* (Monogenea) on *Ceratothoa parallela* (Isopoda) on *Boops boops* (Teleostei)

**DOI:** 10.1051/parasite/2021044

**Published:** 2021-06-07

**Authors:** Chahinez Bouguerche, Fadila Tazerouti, Delphine Gey, Jean-Lou Justine

**Affiliations:** 1 Université des Sciences et de la Technologie Houari Boumediene, Faculté des Sciences Biologiques, Laboratoire de Biodiversité et Environnement : Interactions – Génomes BP 32 El Alia, Bab Ezzouar, Algiers Algeria; 2 Service de Systématique moléculaire, UMS 2700 CNRS, Muséum National d’Histoire Naturelle, Sorbonne Universités 43 rue Cuvier CP 26 75231 Paris Cedex 05 France; 3 UMR7245 MCAM, Muséum National d’Histoire Naturelle 43 rue Cuvier CP 52 75231 Paris Cedex 05 France; 4 Institut Systématique Évolution Biodiversité (ISYEB), Muséum National d’Histoire Naturelle, CNRS, Sorbonne Université, EPHE, Université des Antilles 43 rue Cuvier CP 51 75231 Paris Cedex 05 France

**Keywords:** Hyperparasite, *Cyclocotyla bellones*, Polyopisthocotylea, Isopoda, *Boops boops*, Barcoding

## Abstract

*Cyclocotyla bellones* Otto, 1823 (Diclidophoridae) is a monogenean characterised by an exceptional way of life. It is a hyperparasite that attaches itself to the dorsal face of isopods, themselves parasites in the buccal cavity of fishes. In this study, *Cy. bellones* was found on *Ceratothoa parallela* (Otto, 1828), a cymothoid isopod parasite of the sparid fish *Boops boops* off Algeria in the Mediterranean Sea. We provide, for the first time, molecular barcoding information of a hyperparasitic monogenean, the parasitic crustacean host, and the fish host, with COI sequences.

## Abstract (fr)

*Cyclocotyla bellones* Otto, 1823 (Diclidophoridae) est un monogène caractérisé par un mode de vie exceptionnel. C’est un hyperparasite qui se fixe sur la face dorsale d’isopodes, eux-mêmes parasites dans la cavité buccale de poissons. Dans cette étude, *Cy. bellones* a été trouvé sur *Ceratothoa parallela* (Otto, 1828), un isopode cymothoïdé parasite du poisson sparidé *Boops boops* au large de l’Algérie, en mer Méditerranée. Nous fournissons, pour la première fois, des informations de codes-barres moléculaires d’un monogène hyperparasite, de l’hôte crustacé parasite et de l’hôte poisson, avec des séquences de COI.

## Introduction

*Cyclocotyla bellones* Otto, 1823 is a diclidophorid Monogenea, hyperparasite of cymothoid isopods of the buccal cavity of the sparid fish *Boops boops* (Linnaeus, 1758). It was first described in one of the earliest accounts on monogeneans by Otto (1823), who erected the genus *Cyclocotyla* Otto, 1823 for *Cy. bellones* collected from the skin of the dorsal face of the garfish *Belone belone* (Linnaeus, 1760) off Naples, Italy [62]. It was then frequently reported on isopods parasitic of *B. boops* and of *Spicara* spp. (see Table 1), with a single record on cymothoids of the carangid *Trachurus trachurus* (Linnaeus, 1758) [23].

**Table 1:** Hosts and localities of *Cyclocotyla bellones* reported in the literature.

Host/locality	Reference
*Belone belone* (type host)	
Italy, Mediterranean Sea	[62]
*Ceratothoa oestroides*, buccal cavity of *Trachurus trachurus*	
Spain, Atlantic Ocean	[23]
*Ceratothoa oestroides*, buccal cavity of *Boops boops*	
Monaco, Mediterranean Sea	[22]
France, Mediterranean Sea	[68]
Cymothoids, buccal cavity of *Spicara maena*	
France, Mediterranean Sea	[46]
*Ceratothoa oestroides*, buccal cavity of *Spicara maena*	
Turkey, Mediterranean Sea	[74]
France, Mediterranean Sea	[25]
Spain, Mediterranean Sea	[14]
Algeria, Mediterranean Sea	[5]
*Emetha audouini*, buccal cavity of *Spicara maena*	
Montenegro, Mediterranean Sea	[74]
France, Mediterranean Sea	[25]
*Emetha audouini*, buccal cavity of *Spicara smaris*	
Montenegro, Mediterranean Sea	[66]
France, Mediterranean Sea	[25]
Cymothoidae, *Spicara smaris*	
Montenegro, Mediterranean Sea	[66]
*Boops boops*, Cymothoids	
France, Mediterranean Sea	[46, 59]
*Boops boops*, *Ceratothoa oestroides*	
France, Mediterranean Sea	[25]
Italy, Mediterranean Sea	[53]
*Boops boops, E. audouini*-*Ceratothoa oxyrrhynchaena*-*Ceratothoa parallela*	
France, Mediterranean Sea	[25]
*Boops boops*, Cymothoids	
France, Mediterranean Sea	[46]
*Boops boops*, *Ceratothoa parallela*	
Turkey, Mediterranean Sea	[74]
France, Mediterranean Sea	[68]
Algeria, Mediterranean Sea	Present study

Similarly, a few other diclidophorids were described from parasitic crustaceans: *Allodiclidophora squillarum* (Parona & Perugia, 1889) [syn. *Mesocotyle squillarum* Parona & Perugia, 1889] on the bopyrid *Bopyrus squillarum* Latreille, 1802 off Italy [63]; *Allodiclidophora charcoti* (Dollfus, 1922) [syn. *Cyclobothrium charcoti* Dollfus, 1922] on a female *Ceratothoa oestroides* (Risso, 1816) from the buccal cavity of *T. trachurus* (Linnaeus, 1758) and of *B. boops* (Linnaeus, 1758), caught from Spain and Monaco, respectively [22–24]; and *Diclidophora merlangi* (Kuhn, 1829) Krøyer, 1838 [syn. *Dactylocotyle merlangi* Kuhn, 1829] collected on *Ceratothoa oestroides* from the buccal cavity of *B. boops* from Italy (Taschenberg 1879 *in* [23]).

The identity of the hyperparasite monogenean, the crustacean parasite-host, or the fish host was not confirmed via DNA sequencing in any of these instances. Hence, as part of an ongoing effort to characterize the parasite biodiversity of fishes off the Southern shores of the Mediterranean Sea [3, 6–12, 15–18, 20, 42], molecular characterization of the three members of the hyperparasite-parasite-host association is provided for the first time.

We do not show detailed illustrations or measurements of monogeneans in this paper because these will be provided in a future article; however, we provide a general illustration and a short description.

## Materials and methods

### Collection and sampling of fishes

From 2017 to 2019, 624 specimens of *B. boops* and 45 *Pagellus acarne* (Risso, 1827) were collected from fish markets off Réghaia on the Algerian coast or directly from local fishermen in Bouharoun (36°37′ N, 2°39′ E). Fish specimens were transferred to the laboratory shortly after capture and identified using keys [26] and examined fresh on the day of purchase. The buccal cavities were carefully examined for isopods. Gill arches were also resected and placed in separate Petri dishes containing filtered sea water. The buccal cavity, isopods and isolated gills were observed under a dissecting microscope for the presence of monogeneans.

### Collection of isopods and monogeneans

For the host fish *B. boops*, all isopods (sometimes with visible diclidophorid monogeneans) were removed from the buccal cavity using dissecting forceps. Monogeneans were isolated from the isopods with a fine dissecting needle. Other diclidophorids (*Choricotyle* sp.) parasitic on gills of another sparid, *Pagellus acarne* were removed from the gills using a fine dissecting needle (Table 2). Note that amongst the polyopisthocotyleans collected on *P. acarne*, only *Choricotyle* sp. was included as it is remarkably similar in morphology to species of C*yclocotyla*; *Choricotyle* sp. is a parasite of the fish so no isopod was involved in this particular association.

**Table 2:** Fishes, Isopoda, Monogenea, and their COI sequences. To ensure full traceability and respect of host-parasite relationships, for *Cyclocotyla bellones* one monogenean was collected from one parasitic isopod and each fish, isopod and monogenean individuals were sequenced. *Choricotyle chrysophryi* is a parasite of the fish, so no isopod was involved. All vouchers were deposited in the MNHN.

Fish species	Fish ID	GenBank fish COI sequence	Isopoda species	Isopoda ID	GenBank Isopoda COI sequence	Voucher	Monogenea species	Monogenea ID	GenBank Monogenea COI sequence	Voucher slide
*Boops boops*	Bobo Br2	MZ127221	*Ceratothoa parallela* (female)	Bobo Br2 Iso1	MZ127227	MNHN-IU-2016-9111	*Cyclocotyla bellones*	Bobo Br2 Iso1 MO01	MZ127220	MNHN HEL1307
*Boops boops*	Bobo Br5	MZ127226	*Ceratothoa parallela* (female)	Bobo Br5 Iso1	MZ127219	MNHN-IU-2016-9112	*Cyclocotyla bellones*	Bobo Br5 Iso1 MO01	MZ127224	MNHN HEL1308
*Boops boops*	Bobo Br6	MZ127217	*Ceratothoa parallela* (female)	Bobo Br6 Iso1	MZ127225	MNHN-IU-2016-91123	*Cyclocotyla bellones*	Bobo Br6 Iso1 MO01	MZ127218	MNHN HEL1309
*Pagellus acarne*	PaacaBr1	MZ127223					*Choricotyle* cf. *chrysophryi*	Paaca Br1 MO01	MZ127216	MNHN HEL1310
							*Choricotyle* cf. *chrysophryi*	Paaca Br1 MO02	MZ127222	MNHN HEL1311

### Morphological methods

Monogeneans were preserved in 70% ethanol, stained with acetic carmine, dehydrated in a graded series of alcohol for 15 min each: (70, 96 and 100%), cleared in clove oil, and finally mounted in Canada balsam. Monogeneans were identified on stained whole mounts. Isopods were identified with the help of Prof. Jean-Pierre Trilles (University of Montpellier, France).

### Deposition of specimens

Voucher specimens of monogeneans (hologenophores sensu Pleijel [64]) were deposited in the Muséum National d’Histoire Naturelle, Paris, France (MNHN), under registration numbers MNHN HEL1307–1336. Voucher specimens of isopods (hologenophores) were deposited in the Muséum National d’Histoire Naturelle, Paris, France (MNHN), under registration numbers MNHN-IU-2016-9111–9113. Fish specimens were not deposited.

### Molecular methods

For complete traceability of the molecular study, special care was taken to ensure that hosts and monogeneans were labelled with respect to host-parasites relationships [3, 7, 8, 10, 11, 39].

For three individual *B. boops*, the parasitic female isopod and one monogenean on this individual isopod were extracted (Table 2). A tissue sample from the gill of the fish was taken and a pereopod was detached from each infected isopod and submitted to molecular analysis. For the monogenean, a small lateral part of the body was separated with a scalpel and submitted to molecular analysis, and the rest of the body was mounted on a slide as a voucher for drawing and deposition in a Museum collection. This ensures that the molecular identification of the host fish, the parasitic isopod and their monogenean parasite correspond perfectly at the individual host and parasite levels, and enable morphological assessment of sequenced monogeneans. Slides of monogeneans were deposited in the Muséum National d’Histoire Naturelle, Paris, France (MNHN), under registration numbers MNHN HEL1307–1309.

### Molecular barcoding of fish

Total genomic DNA was isolated using a QIAamp DNA Mini Kit (Qiagen, Courtaboeuf, France), according to the manufacturer’s instructions. The 5′ region of the mitochondrial cytochrome c oxidase subunit I (COI) gene was amplified with the primers TelF1 (5′–TCGACTAATCAYAAAGAYATYGGCAC–3′) and TelR1 (5′–ACTTCTGGGTGNCCAAARAATCARAA–3′) [21]. PCR reactions were performed in 20 μL, containing 1 ng of DNA, 1× CoralLoad PCR buffer, 3 mM MgCl_2_, 66 μM of each dNTP, 0.15 μM of each primer, and 0.5 units of Taq DNA polymerase (Qiagen). The amplification protocol was 4 min at 94 °C, followed by 40 cycles at 94 °C for 30 s, 48 °C for 40 s, and 72 °C for 50 s, with a final extension at 72 °C for 7 min. PCR products were purified (Ampure XP Kit, Beckman Coulter, Brea, CA, USA) and sequenced in both directions on a 3730 × l DNA Analyzer 96-capillary sequencer (Applied Biosystems, Foster City, CA, USA). We used CodonCode Aligner version 3.7.1 software (Codon Code Corporation, Dedham, MA, USA) to edit sequences, compared them to the GenBank database content with BLAST, and deposited them in GenBank (accession numbers in Table 1). Species identification was confirmed with the BOLD identification engine [67].

### Molecular barcoding of isopods

Total genomic DNA from a pereopod was isolated using a QIAamp DNA Mini Kit (Qiagen, Courtaboeuf, France), according to the manufacturer’s instructions. The 5′ region of the COI gene was amplified with the “universal” primers LCO1490 (5′–GGTCAACAAATCATAAAGATATTGG–3′) and HCO2198 (5′–TAAACTTCAGGGTGACCAAAAAATCA–3′) [27]. PCR reactions were performed in 20 μL, containing 1 ng of DNA, 1× CoralLoad PCR buffer, 3 mM MgCl_2_, 66 μM of each dNTP, 0.15 μM of each primer, and 0.5 units of Taq DNA polymerase (Qiagen). The amplification protocol was 4 min at 94 °C, followed by 40 cycles at 94 °C for 30 s, 48 °C for 40 s, and 72 °C for 50 s, with a final extension at 72 °C for 7 min. Sequences were obtained as for fish and were deposited in GenBank (accession numbers in Table 1).

### Molecular barcoding of monogeneans

Total genomic DNA was isolated using a QIAmp DNA Micro Kit (Qiagen). The specific primers JB3 (=COIASmit1) (forward 5′–TTTTTTGGGCATCCTGAGGTTTAT–3′) and JB4.5 (=COI-ASmit2) (reverse 5′–TAAAGAAAGAACATAATGAAAATG–3′) were used to amplify a fragment of 424 bp of the COI gene [13, 48]. PCR reactions were performed in 20 μL, containing 1 ng of DNA, 5× iProof HF buffer, 0.25 mM dNTP, 0.15 μM of each primer, and 0.5 units of iProof HF DNA polymerase (Bio-Rad). Thermocycles consisted of an initial denaturation step at 94 °C for 2 min, followed by 37 cycles of denaturation at 94 °C for 30 s, annealing at 48 °C for 40 s, and extension at 72 °C for 50 s. The final extension was conducted at 72 °C for 5 min. Sequences were obtained as for fish and were deposited in GenBank (accession numbers in Table 1).

### Trees and distances

For fishes, the phylogenetic analyses included three sequences of *B. boops* and one *Pagellus acarne* generated in this study, and other sequences of these fishes available in GenBank (Table 3), whilst *Spicara maena* (Linnaeus, 1758), a sparid previously reported as a host of *Cy. bellones* [5] was used as an outgroup.

**Table 3:** Accession numbers of COI sequences used in the molecular analysis of fishes. Species previously reported as hosts of *Cyclocotyla bellones* or as hosts for isopods bearing the latter are in bold. Note that all hosts are sparids. *Choricotyle chrysophryi* is a parasite of the fish, so no isopod was involved. *, new sequences.

Host species	Origin	GenBank	Source
*Boops boops*	Algeria	MZ127221*	Present study
*Boops boops*	Algeria	MZ127226*	Present study
*Boops boops*	Algeria	MZ127217*	Present study
*Boops boops*	Algeria	MK317921	[8]
*Boops boops*	Algeria	MT666082–MT666086	[11]
*Boops boops*	Italy	KJ709490	[47]
*Boops boops*	Malta	KJ709712	[47]
*Boops boops*	Turkey	KC500341–KC500342	[40]
*Boops boops*	Turkey	KC500351	[40]
*Boops boops*	Mediterranean Sea	KJ012295–KJ012296	[2]
*Boops boops*	Portugal	JQ774986–JQ774987	[19]
*Boops boops*	Eastern Mediterranean	KJ012383–KJ012385	[2]
*Pagellus acarne*	Algeria	MZ127223*	Present study
*Pagellus acarne*	Italy	KJ709574	[47]
*Pagellus acarne*	Portugal	JQ775093–JQ775092	[19]
*Pagellus acarne*	Spain	FN689213	[44]
*Pagellus acarne*	Turkey	FN689212	[44]
*Spicara maena*	Portugal	KJ768312	[47]

For isopods, the molecular analysis was based upon mouth dwelling cymothoids, mainly species previously reported as hosts of *Cy. bellones* (Table 4). The body surface isopod *Anilocra clupei* Williams & Bunkley-Williams, 1986 was used as an outgroup.

**Table 4:** Accession numbers of COI sequences used in the molecular analysis of cymothoids. All *Ceratothoa* species infect the buccal cavity of their fish host, whereas individuals of *Anilocra clupei* infect the body surface of the fish. Species previously reported as hosts of *Cyclocotyla bellones* or as hosts for crustaceans bearing the latter are in bold. S, Sparidae. M, Moronidae. C, Carangidae. Cl, Clupeidae. *, new sequences.

Parasite species	Host species	Origin	GenBank	Source
***Ceratothoa parallela***	***Boops boops* (S)**	Mediterranean Sea (Algeria)	MZ127227*	Present study
***Ceratothoa parallela***	***Boops boops* (S)**	Mediterranean Sea (Algeria)	MZ127219*	Present study
***Ceratothoa parallela***	***Boops boops* (S)**	Mediterranean Sea (Algeria)	MZ127225*	Present study
*Ceratothoa collaris*	*Lithognathus mormyrus* (S)	Mediterranean Sea (Tyrrhenian Sea)	EF455816	[41]
*Ceratothoa* sp.	*Dentex gibbous* (S)	Atlantic Ocean (West Africa)	LC159551	[33]
***Ceratothoa oestroides***	*Sparus aurata* (S)	Mediterranean Sea (Adriatic).	GQ240266	[58]
***Ceratothoa oestroides***	*Sparus aurata* (S)	Mediterranean Sea (Adriatic)	GQ240267	[58]
***Ceratothoa oestroides***	***Boops boops* (S)**	Mediterranean Sea (Adriatic)	GQ240272	[58]
***Ceratothoa oestroides***	***Boops boops* (S)**	Mediterranean Sea (Adriatic)	GQ240273	[58]
***Ceratothoa oestroides***	*Dicentrarchus labrax* (M)	Mediterranean Sea (Adriatic)	GQ240276	[58]
***Ceratothoa oestroides***	*Dicentrarchus labrax* (M)	Mediterranean Sea (Adriatic)	GQ240277	[58]
***Ceratothoa oxyrrhynchaena***	*Dentex hypselosomus* (S)	Pacific Ocean (Japan)	LC159545	[33]
***Ceratothoa oxyrrhynchaena***	*Dentex abei* (S)	Pacific Ocean (Japan)	LC160310	[33]
*Ceratothoa verrucosa*	*Pagrus major* (S)	Pacific Ocean (Japan)	LC159556	[33]
*Ceratothoa verrucosa*	*Evynnis tumifrons* (S)	Pacific Ocean (Japan)	LC160317	[33]
*Anilocra clupei*	*Sardinella zunasi* (C)	Pacific Ocean (Japan)	LC159540	[33]
*Anilocra clupei*	*Etrumeus micropus* (Cl)	Pacific Ocean (Japan)	LC160309	[33]

For monogeneans, most sequences of Diclidophoridae available in GenBank were included in the phylogenetic analysis (Table 5), with three sequences of *Cy. bellones* and one of *Choricotyle* cf. *chrysophryi* obtained in the present study. A sequence of *Plectanocotyle gurnardi* (Van Beneden & Hesse, 1863), a member of Plectanocotylidae Monticelli, 1903, grouped with the Diclidophoridae in a previous phylogeny of Monogenea [38], was used as an outgroup. Molecular analyses were performed in MEGA, version 7 [45]. The trees were inferred using the neighbour joining (NJ) method [69] and the maximum likelihood (ML) method using MEGA7 [45]. Based on the best model, maximum likelihood was used for the best fitting tree according to the Hasegawa-Kishino-Yano with gamma distribution and invariant sites (HKY + G) for fishes; Tamura 3-parameter with gamma distribution and invariant sites (T92 + I) for isopods, and Tamura 3-parameter with gamma distribution (T92 + G) for monogeneans. The robustness of the inferred analysis was assessed using a bootstrap procedure with 1000 replications. Genetic distances, *p*-distance and Kimura-2 parameter distance (K-2-P), [43] were estimated with MEGA7 and all codon positions were used.

**Table 5:** Accession numbers of COI sequences used in the molecular analysis of diclidophorid monogeneans. *, new sequences.

Parasite species	Host species	Origin	GenBank	Source
*Cyclocotyla bellones*	*Ceratothoa parallela* from *Boops boops* (Cymothoidae/ Sparidae)	Mediterranean Sea (Algeria)	MZ127220*	Present study
*Cyclocotyla bellones*	*Ceratothoa parallela* from *Boops boops*	Mediterranean Sea (Algeria)	MZ127224*	Present study
*Cyclocotyla bellones*	*Ceratothoa parallela* from *Boops boops*	Mediterranean Sea (Algeria)	MZ127218*	Present study
*Choricotyle* cf. *chrysophryi*	*Pagellus acarne* (Sparidae)	Mediterranean Sea (Algeria)	MZ127216*	Present study
*Choricotyle* cf. *chrysophryi*	*Pagellus acarne* (Sparidae)	Mediterranean Sea (Algeria)	MZ127222*	Present study
*Choricotyle anisotremi*	*Anisotremus scapularis* (Haemulidae)	Pacific Ocean (Chile)	KJ794206	[60]
*Choricotyle anisotremi*	*Anisotremus scapularis*	Pacific Ocean (Chile)	KJ794207	[60]
*Neoheterobothrium affine*	*Paralichthys dentatus* (Paralichthyidae)	Atlantic Ocean (USA)	AB478782	[75]
*Neoheterobothrium hirame*	*Paralichthys olivaceus* (Paralichthyidae)	Pacific Ocean (Japan)	AB162615	[75]
*Parapedocotyle prolatili*	*Prolatilus jugularis* (Pinguipedidae)	Pacific Ocean (Chile)	KJ794219	[60]
*Parapedocotyle prolatili*	*Prolatilus jugularis*	Pacific Ocean (Chile)	KJ794218	[60]
*Pedocotyle bravoi*	*Stellifer minor* (Sciaenidae)	Pacific Ocean (Chile)	KJ794210	[60]
*Pedocotyle bravoi*	*Stellifer minor*	Pacific Ocean (Chile)	KJ794210	[60]
*Plectanocotyle gurnardi*	*Chelidonichthys lastoviza* (Triglidae)	Mediterranean Sea (Algeria)	MK275654	Ayadi et al. (unpublished)

BLAST analyses of the COI sequences of monogeneans, isopods and fishes obtained in the present study were performed using NCBI and BOLD databases [67].

## Results

### Details on parasitism

*Boops boops*, parasitism by *Ceratothoa parallela*. Prevalence and intensity: 14% (93 out of 624 fish), up to 2 cymothoids/fish.

*Ceratothoa parallela*, parasitism by *Cyclocotyla bellones*. Prevalence and intensity: 11% (10 out of 93 isopods), up to 2 monogeneans/cymothoids.

No *Pagellus acarne* were infected with cymothoids. All monogeneans were collected from the fish gills.

### Short description of *Cyclocotyla bellones* Otto, 1823 (Fig. 1)

Type-host: *Belone belone* (Linnaeus, 1760), garfish (Belonidae Bonaparte, 1835).

**Figure 1:**
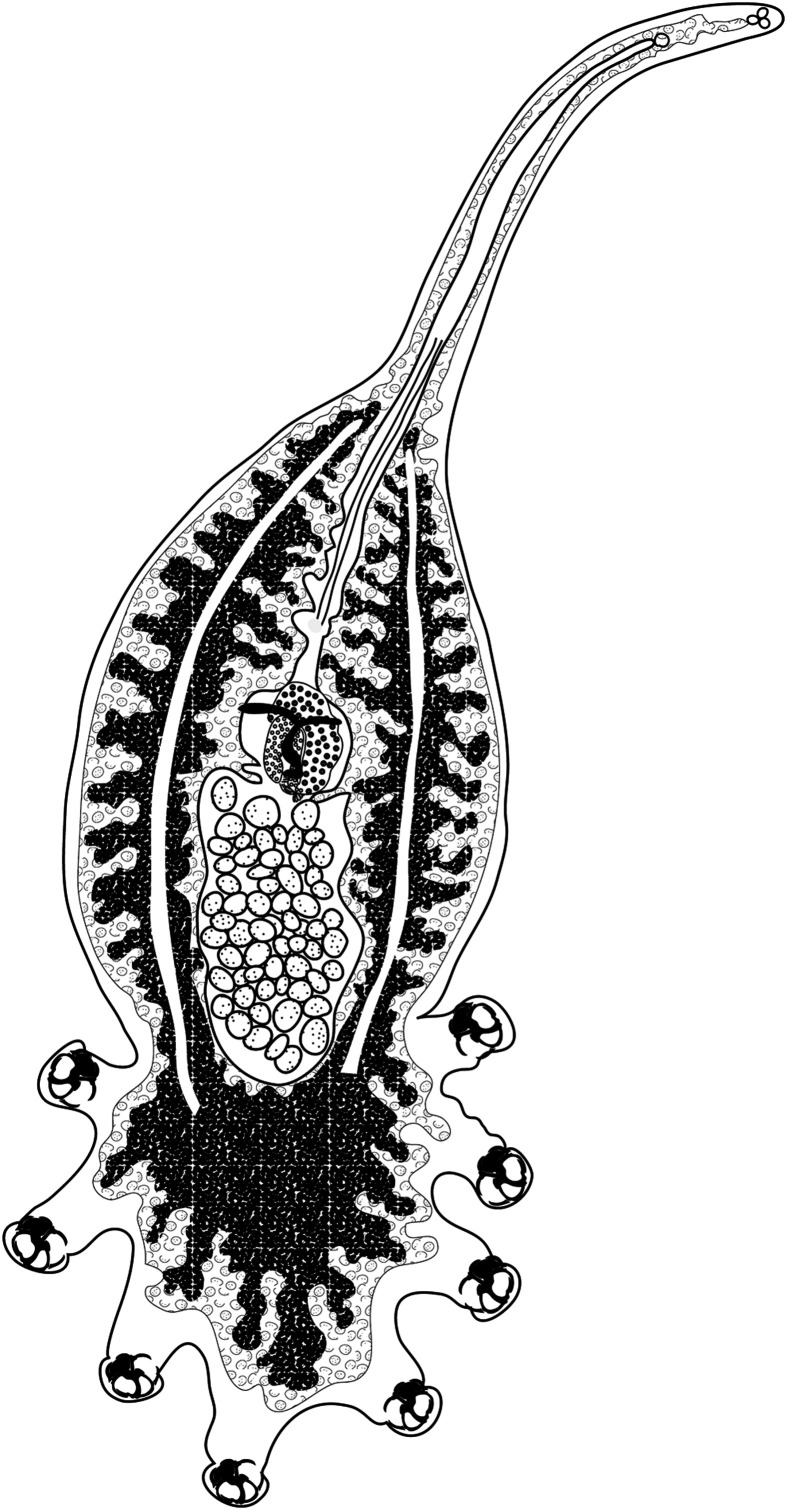
*Cyclocotyla bellones* Otto, 1823. Whole body, MNHN HEL1312.

Additional hosts: *Bopyrus squillarum* Latreille, 1802 (Bopyridae Rafinesque, 1815). Isopods of *Spicara maena* (Linnaeus, 1758) (Sparidae Rafinesque, 1818), the blotched picarel; of *Spicara smaris* (Linnaeus, 1758), the picarel; and of *B. boops* (Linnaeus, 1758) (Sparidae), the bogue. *Ceratothoa parallela* (Otto, 1828) (Cymothoidae Leach, 1818) from *B. boops* (this paper).

Type-locality: Italy [62].

Additional localities: Montenegro, France, and Turkey. Off Bouharoun (36° 37′ 24″ N, 2° 39′ 17″ E), off the Algerian coast (this paper).

Specimens from Algeria, from *Ceratothoa parallela* (Cymothoidae) of buccal cavity. Vouchers deposited in the collection of the Muséum National d’Histoire Naturelle, Paris (MNHN HE1307–HEL1326). Vouchers with molecular information, three specimens mounted on slide, a small lateral part cut off and used for molecular analysis. (MNHN HEL1307, GenBank MZ127220; MNHN HEL1308, GenBank MZ127224; MNHN HEL1309, GenBank MZ127218).

Body elongate, divided into three regions: a tapered anterior region; an enlarged and rounded middle region and a posterior region formed by the haptor. Haptor ovoid, bearing four pairs of pedunculated clamps. Prohaptoral suckers paired, rounded. Caeca ramified medially and laterally. Testicles post-ovarian, follicular, numerous in intercaecal field. Vas deferens sinuous, extending anteriorly to male copulatory organ. Ovary median, folded. Oötype postovarian, surrounded by mass of Mehlis’ glands. Uterus running along body midline and opening into genital atrium. Seminal receptacle present. Genito-intestinal canal originating from left intestinal branch. Vitelline follicles large, coextensive with intestinal caeca and invading haptor. Vitelloducts Y-shaped. Vagina absent.

### Molecular identification of fish

The provisional identification of fish species using morphological characteristics was confirmed by the DNA barcoding approach. The obtained sequences were 652 bp long. BLAST analyses of the COI sequences of the present study with NCBI and BOLD databases showed sequence similarity values of 100% for *B. boops* and 99.85 % for *P. acarne*.

**Figure 2:**
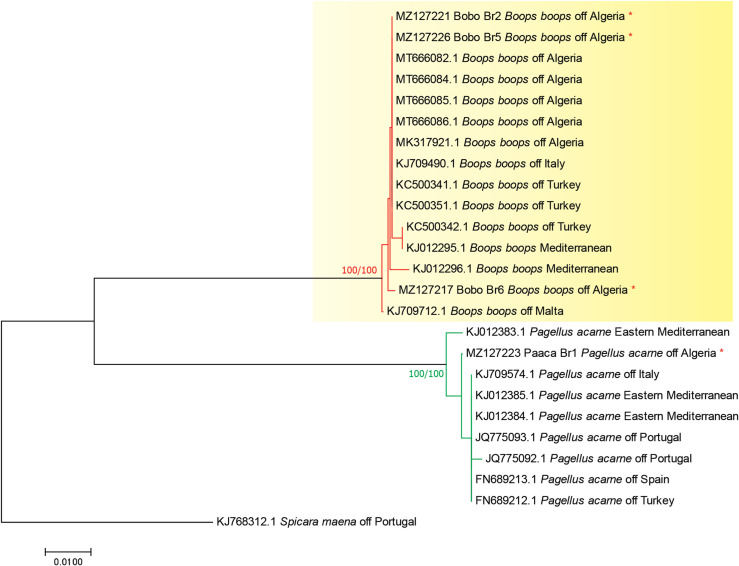
Molecular phylogenetic analysis based on maximum likelihood, inferred from COI sequences of *Boops boops* in relation to other hosts of *Cyclocotyla bellones*. The tree is drawn to scale, with branch lengths measured in the number of substitutions per site.

The ML tree is shown in Figure 2. The newly generated sequences of *B. boops* clustered in a well-supported clade (100% bootstrap). All sequences of *P. acarne*, including our newly generated sequence, clustered in a single robust clade.

Distances were computed using Kimura 2-parameter distance and p-distance. Our sequences of *B. boops* were identical (0% intraspecific variation). All Sequences of *B. boops* (available in GenBank plus our newly generated sequences) showed little to no variation (0–1%). The divergence among sequences of *P. acarne* was also low, and ranged between 0 and 1%.

### Molecular information on isopods

Isopods were identified as *Ceratothoa parallela* (Otto, 1828). The newly acquired sequences were 658 bp long. BLAST analyses of the COI sequences with NCBI and BOLD databases showed sequence similarity values of 80.47–80.63% for “*Ceratothoa* sp.”; however, no sequence identified at the species level was available from the databases.

**Figure 3:**
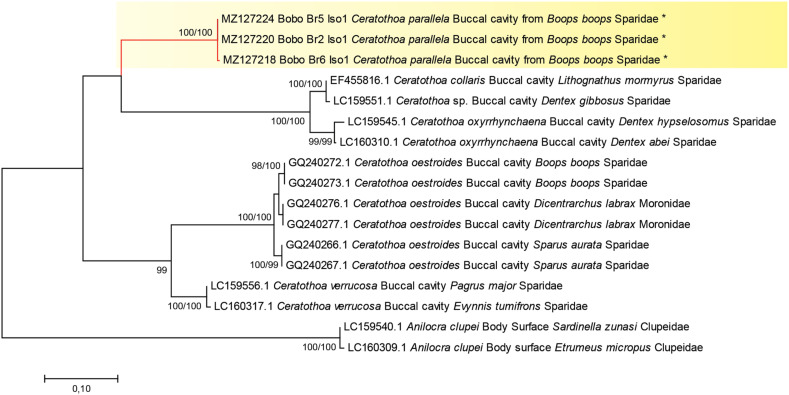
Molecular phylogenetic analysis based on maximum likelihood, inferred from COI sequences of *Ceratothoa parallela* in relation to other cymothoids hosts of *Cyclocotyla bellones*. The tree is drawn to scale, with branch lengths measured in the number of substitutions per site.

Phylogenetic trees were constructed based on our newly generated COI sequences of *Ceratothoa parallela*, and combined datasets of *Ceratothoa* spp., mainly those previously reported as hosts of *C. bellones*. The analysis involved 13 nucleotide sequences, and there was a total of 409 positions in the final dataset. The topologies were nearly consistent among the ML (Fig. 3) and the NJ trees. The “mouth dwelling cymothoid clade” was well separated from the outgroup, which included body surface isopods. The mouth dwelling cymothoids were separated into two clades, one including *C. oestroides* and *C. verrucosa,* and one including *C. parallela*, *C. collaris* and *C. oxyrrhynchaena*.

There was no intraspecific variation among our three sequences of *Ceratothoa parallela*. The mean intraspecies distance of sequences of *Ceratothoa oestroides* from three hosts, *B. boops*, *Sparus aurata* and *Dicentrarchus labrax* ranged between 0% and 2%, suggesting that the same species is harboured by the three fish species. Distances between individuals of congeneric species were high, the highest being between *Ceratothoa oestroides* from *B. boops* and *C. parallela*, 28%.

### Molecular characterisation of monogeneans

**Figure 4:**
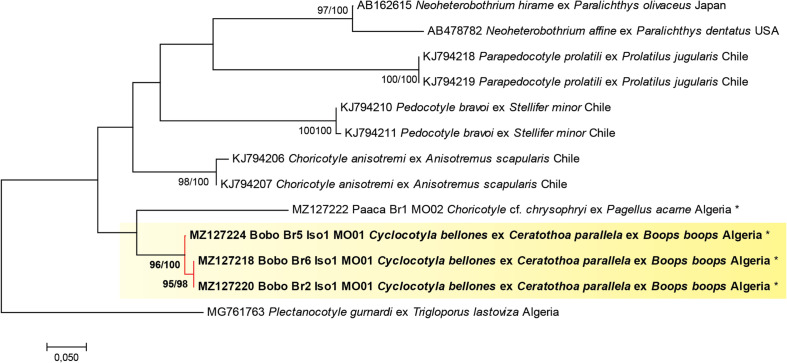
Molecular phylogenetic analysis based on maximum likelihood, inferred from COI sequences of *Cyclocotyla bellones* in relation to other taxa of diclidophorids; a plectanocotylid was chosen as the outgroup. Values along branches indicate percentage bootstrap support for maximum likelihood and neighbour joining methods (ML/NJ). Nodes without bootstrap values had support lower than 50% and were omitted. There were a total of 389 positions in the final dataset. All specimens of *Cyclocotyla bellones* were grouped into a single monophylum and showed little variation (≤1%). The tree is drawn to scale, with branch lengths measured in the number of substitutions per site. ex: from.

The newly generated sequences were 402 bp long. The COI sequences of *Cy. bellones* were aligned with other diclidophorid sequences. For trees, the NJ and ML methods led to similar topologies; we show only the ML tree in Figure 4. The analysis involved 13 nucleotide sequences, and there was a total of 337 positions in the final dataset.

The three sequences of *Cy. bellones* reported in the present study formed a well-supported monophyletic lineage (96 bootstraps in ML, 100 in NJ). The phylogenetic analysis therefore supports *Cy. bellones* as a distinct species from *Choricotyle* cf. *chrysophryi*. The sequence of *Choricotyle* cf. *chrysophryi* nested within a *Cyclocotyla* + *Choricotyle* clade, but *Choricotyle* was paraphyletic. However, support values for most clades were low and this phylogeny is not discussed further.

Distances were computed using Kimura 2-parameter distance and *p*-distance. Sequences of *Cy. bellones* showed little to no variations between them: 0–1%. The differences between sequences of *Cy. bellones* and *Choricotyle* cf. *chrysophryi* from the sparid *P. acarne* were 18% and 16% in K-2-P and *p* distance, respectively. *Cyclocotyla bellones* differed from *Neoheterobothrium hirame*, and two sequences of *Choricotyle anisotremi* by 22% and 18%, respectively (*p* distances were 19% and 16%, respectively). The highest divergence was observed between *Cy. bellones* and *Parapedocotyle prolatili* (26–27% in K-2-P, 22–23% in *p* distance).

## Discussion

### Hyperparasitic monogeneans

Overall, records of hyperparasitic monogeneans are rather scant. Among the Monopisthocotylea Odhner, 1912, this tripartite association is known for two cases: the Udonellidae Taschenberg, 1879, with *Udonella* spp. on parasitic copepods of various fishes [73] and the Capsalidae Baird, 1853 with *Capsala biparasitica* (Goto, 1894) on a copepod on the gills of the tuna *Thunnus albacares* (Bonnaterre, 1788) [28].

Similarly, hyperparasitism in known in only one family of the Polyopisthocotylea Odhner, 1912, the Diclidophoridae, in four genera: *Cyclocotyla* [62], *Allodiclidophora* Yamaguti, 1963 [22, 23, 63, 81], *Choricotyle* Van Beneden & Hesse, 1863 [28, 32, 51] and *Diclidophora* Krøyer, 1838 [22, 23]. Except *Allodiclidophora squillarum* (Parona & Perugia, 1889) reported on the ovigerous lamellae of the bopyrid *Bopyrus squillarum*, a parasite of shrimp, all the previously mentioned hyperparasites were found on cymothoids of the buccal cavity of marine fishes [22, 23, 28, 32, 51]. However, it is noteworthy that the taxonomic status of the previously mentioned diclidophorids should be further examined as several of them were placed in different genera, based on characters of little generic significance, or considered synonyms with little justification [51, 65, 81]. Hence, molecular markers will be indispensable in future studies for the delimitation of genera and species.

Members of the Diclidophoridae Cerfontaine, 1895 are cosmopolitan polyopisthocotyleans, infecting gills, the operculum, and gill cavity of teleosts and elasmobranchs [54]. To date, 51 genera are assigned to this family (WoRMS, 2020). Even though great progress has been made in generating molecular data for members of this family [4, 38, 49, 50, 52, 56, 59-61, 71, 75, 82], amongst 173 species allocated to this family, molecular markers are available for only 24 species. Internal transcribed spacer (ITS) sequences are known for five species [75, 82]. 18S and 28S RNA ribosomal sequences are available for 18 species across 11 genera [4, 38, 49, 50, 52, 56, 59–61, 71]. COI sequences are the least numerous, known for only 10 species across 6 genera [38, 60, 82].

### Cymothoid isopods

The COI divergences observed between the newly generated sequences of *Ceratothoa parallela* sampled from *B. boops* ranged from 0 to 2% and did not exceed the 3% and 5% COI thresholds suggested for species-level divergences [34, 35, 70], confirming the morphological identification of the three specimens as conspecific. These findings can further be strengthened by additional COI sequences of this species from various localities and hosts.

Overall, the Cymothoidae are taxonomically challenging as descriptions of many species were originally based only on the morphology of few or sometimes single specimens, thus providing no information on polymorphism [76]. In addition, many misidentifications and incorrect data on species were generated when not considering the morphological variability ascribed to the parasitic lifestyle of cymothoids, polymorphism and sister species, as well as the variations in attachment site and the morphological adaptations [29, 72, 76]. In recent years, numerous genera and species described early were revised, eliminating some of the uncertainties and confusion [30, 72, 76]. Hence, every effort should be made to generate molecular data and deposit specimens in collections, which can be used subsequently.

*Ceratothoa* is a large genus, with 25 species presently accepted [31]. The species studied here, *Ceratothoa parallela*, was designated in 2015 as the type-species of the genus [55]. Unfortunately, not many COI sequences are available for members of this genus and of the family of Cymothoidae overall, and almost half are from unpublished works. COI sequences are known for 16 genera [33, 36, 41, 57, 77–80]. 16S sequences are available for 15 genera [33, 37, 41, 78, 80]. 18S sequences are known for 4 genera (all from unpublished works) and 28S sequences are available for only 3 genera [33]. The complete mitochondrial genome is known for 2 genera of Cymothoidae from unpublished works, available only in GenBank: *Asotana* Schioedte & Meinert, 1881 (Zou et al.) and *Ichthyoxenos* Herklots, 1870 (Hua et al.) (GenBank MZ127218, MK790137).

### Cyclocotyla bellones

Genetic variations among our newly generated sequences of *Cy. bellones* were very low, 0–1%, a divergence lower than interspecific distances found in other studies of polyopisthocotylean monogeneans (1% vs. 10.2–15.0%; [7]). The divergence between sequences of *Cy. bellones* and *Choricotyle* cf. *chrysophryi* from the sparid *P. acarne* reached 18% and 16% in K-2-P and *p* distance, respectively, well above the 3% species threshold often admitted for Monogenea [1], and thus the separation between the two species is confirmed genetically.

In the present study, *Cy. bellones* was most exclusively on the isopods, mainly on the upper part of the pereon. These results agree with Euzet & Trilles (1961) who found *Cy. bellones* often attached to the crustacean (telson, pleon, rarely perion) and exceptionally in the buccal cavity of the fish, the palate and the internal edge of the upper lip [25]. We found a single specimen unattached in a Petri dish containing gills and isopods. We carefully examined the gills of *B. boops* during our four-year survey [6, 8, 11], but we have not observed any *Cy. bellones* attached to gills nor in the buccal cavity. Hence, we are confident that this monogenean attaches itself to the isopod and not the fish.

*Cyclocotyla bellones* has been recorded on several hosts and from different localities (Table 1). It has been reported on cymothoids of the oral cavity of four fish species, *B. boops*, *Spicara maena* (Linnaeus, 1758), *S. smaris* (Linnaeus, 1758) and *T. trachurus*. Distributions of the previously mentioned host species overlap as all hosts co-exist in the Mediterranean Sea or in the Eastern Atlantic Ocean. The parasitic isopods have a larger distribution extending to the northern Indo-pacific and coexist with other fish hosts in the Black Sea, Mediterranean and Eastern Atlantic. Therefore, while we took a conservative position and tentatively identified the diclidophorid from cymothoids on *B. boops* as *Cy. bellones*, it is not unlikely that this hyperparasite is a species complex.

## Conflict of interest

The Editor-in-Chief of Parasite is one of the authors of this manuscript. COPE (Committee on Publication Ethics, http://publicationethics.org), to which Parasite adheres, advises special treatment in these cases. In this case, the peer-review process was handled by an Invited Editor, Jérôme Depaquit.
